# Is personality a driving force for socioeconomic differences in young adults’ health care use? A prospective cohort study

**DOI:** 10.1007/s00038-016-0927-4

**Published:** 2016-11-30

**Authors:** Maren Kraft, Koos Arts, Tanja Traag, Ferdy Otten, Hans Bosma

**Affiliations:** 1 Department of Social Medicine, School for Public Health and Primary Care (CAPHRI), Maastricht University, UM/CAPHRI, PO Box 616, 6200 MD Maastricht, The Netherlands; 2 Statistics Netherlands, Centraal Bureau voor de Statistiek, PO Box 4481, 6401 CZ Heerlen, The Netherlands

**Keywords:** Socioeconomic health inequalities, Personality, Individual differences, Indirect selection

## Abstract

**Objectives:**

To relate personality characteristics at the age of 12 to socioeconomic differences in health care use in young adulthood. And thereby examining the extent to which socioeconomic differences in the use of health care in young adulthood are based on differences in personality characteristics, independent of the (parental) socioeconomic background.

**Methods:**

Personality of more than 13,000 Dutch 12-year old participants was related to their health and socioeconomic position after a follow-up of 13 years (when the participants had become young adults).

**Results:**

In young adulthood, low socioeconomic status was related to high health care use (e.g. low education -hospital admission: OR = 2.21; low income -GP costs: OR = 1.25). Odds ratios (for the socioeconomic health differences) did not decrease when controlled for personality.

**Conclusions:**

In this Dutch sample of younger people, personality appeared not to be a driving force for socioeconomic differences in health care use. Findings thus do not support the personality-related, indirect selection perspective on the explanation of socioeconomic differences in health.

## Introduction

The dominating view in social epidemiology is that socioeconomic status causes differences in material circumstances, health behaviours, and psychological attributes which in turn cause differences in health (Borell et al. 2013; Mackenbach 2015; Whitehead 1998). Recent literature (Chapman et al. 2011; Mackenbach 2012; Marmot et al. 1997), including a report on British Household Panel Survey data (De Vries and Rentfrow 2016), however, indicate the importance of individual characteristics for later socioeconomic position and later health. Researchers in the field thereby point to the understudied possibility of third factors underlying socioeconomic attainment, future health, and the association of low socioeconomic status with poor health. Personality traits might be such underlying factors.

A personality trait, such as conscientiousness, for example, has been found protective against smoking and its related diseases and it has also been found predictive of long-term career success (Judge et al. 1999; Mackenbach 2015; Pluess and Bartley 2014). There is further evidence that personality might have an impact on the school career and processes related to social mobility (Mackenbach 2005; Traag 2012). Policies for public health interventions can learn from such evidence, as interventions inspired by evidence on a fundamental role for personality will look different than those inspired by the view that dominates social epidemiology.

However, not many studies have explicitly addressed to what extent personality is an underlying driving force (Chapman et al. 2011; Deary et al. 2010). Furthermore, prior research is often unable to have a personality measurement prior to the measurement of the outcomes which complicates conclusions on causality (Chapman et al. 2009; Nabi et al. 2008; Van Bon-Martens et al. 2012). Whereas some studies found that personality explained some of the social gradient in mortality in men but had little explaining power in women (Nabi et al. 2008), others found personality accounted for 20% of the risk in men and women with lower socioeconomic status (Chapman et al. 2009). In addition, no effect has been found for Type D personality on the risk for low socioeconomic status (Van Bon-Martens et al. 2012). The timing of measurements, however, should be considered as important for the interpretation of the examined pathways (Singh-Manoux 2005). Last, in large studies it has been challenging to find a personality measurement tool that at the same time is time-efficient and less costly (Gallacher 2008; Roberts et al. 2007).

**Fig. 1:**
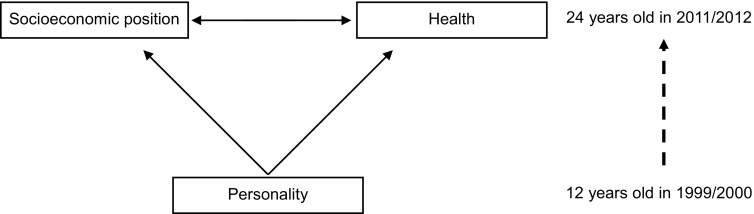
Working model of the association between personality in 1999 (at 12 years old) and socioeconomic differences in health care use in 2011/2012 (at 24 years old). The Netherlands, 1999–2012

Hence, using data on more than 13,000 twelve-year old Dutch participants who from 12 year onwards were followed up for their socioeconomic and health-related life course outcomes until the age of 24, we set out to examine whether and how personality traits predict socioeconomic differences in health care use in young adulthood (Fig. 1).

## Methods

### Study population

The Secondary Education Pupil Cohort 1999 (VOCL’99) started in school year 1999/2000 as a prospective cohort study carried out by Statistics Netherlands (CBS) and the Groningen Institute for Education Research (GION). The 12-year old participants, visiting the first year of secondary education, were followed up for 13 years, until the end of 2012. A random sample of 246 schools was asked to participate, from which 126 secondary schools responded. This resulted in a nationally representative sample of 19,391 Dutch pupils (Kuyper et al. 2003; Traag 2012). Children and parents filled in questionnaires at baseline. Data on socioeconomic position and health until the end of 2012 have been linked to the VOCL’99 cohort using national registers as maintained by CBS. After exclusion of missing cases due to death (*N* = 58), nonresponse on the personality questionnaire (*N* = 4387) and missing covariates (*N* = 2014), 12,932 participants (67%) remained for analyses.

### Measures

The variables have been assessed through both, written questionnaires at baseline and by linking national data registers to the VOCL’99 cohort. The linking procedure needed several steps. First, linking data to the VOCL’99 cohort, gender, date of birth and the postcode of participants were used as keys. Second, using these keys, the participants were linked uniquely to the Dutch municipal population register (GBA). Third, in the final step, the linking to the national registers regarding health care use and socioeconomic position could take place. For every successful link, a unique record identification numbers (RIN) was created (Willenborg and Heerschap 2012). The success rate linking was 99.8% for the participants and 99.3% for the mothers (regarding the parental income measure).

### Health care use

Four health care use endpoints have been used. Hospital admissions (no, yes) were available through the National Medical Registration [Landelijke Medische Registratie (LMR)] in 2011 (2012 data were not sufficiently reliable and were therefore not used) (De Bruin et al. 2003). The LMR derives from Dutch Hospital Data (DHD) and includes all academic, general and categorical hospitals, with the exception of centres for rehabilitation, asthma, and epilepsy. Deliveries without complications, part-time treatment for psychiatric illnesses, and day-time rehabilitation treatment were not registered. Only one of the two categorical cancer clinics participated. The coverage is about 84%. Health care costs in 2012 were also linked to the VOCL’99 cohort. To the extent that these services are covered by the Dutch basic insurance, these costs relate to the use of services of general practitioners and hospitals as these are actually reimbursed by the health insurance companies [Zorgverzekeringswet (ZVW)] (Statistics Netherlands 2015a). The GP costs include registration fees, consultancy costs and other costs made by the GP for the particular patient. Hospital costs are defined as costs made by medical specialised care in the hospital. The costs were summed and subsequently dichotomised into 80% with the lowest costs and 20% with the highest costs. Medication use in 2012 was also linked to the VOCL’99 cohort. The data included reimbursed medicines under the Dutch statutory basic medical insurance (College voor Zorgverzekering, CvZ) (Statistics Netherlands 2015b). For our purposes, we examined any use of medicines versus no use.

### Socioeconomic outcomes

Socioeconomic outcomes in 2012 were assessed by two variables. First is the highest attained level of education which can range from primary education to university education (13 ordinal categories, Standaard Onderwijsindeling (SOI); Statistics Netherlands 2015c). It represents the highest educational level for which participants received a certificate. Second, the 2012 household income of participants, which was available from the integral Dutch Tax Administration, was linked. The household income equivalised for both composition of the household and the number of household members indicated to what percentile of the Dutch households’ income distribution participants could be assigned. Both socioeconomic outcomes were recoded into thirds (using tertiles).

### Personality

The Five Factor Personality Inventory (FFPI) measured participants’ scores on five personality traits (conscientiousness, extraversion, agreeableness, emotional stability and openness to experience) in 2000. The FFPI includes 100 response items, measuring each personality trait on a five-point scale, ranging from 1 = not at all applicable to 5 = entirely applicable. The inventory has been proven valid and reliable for young adults (Hendriks et al. 2008). The FFPI scoring software applied algorithms to assign scores to participants. Using tertiles, the personality scores were categorised into a high, medium, and low scoring group.

### Covariates

Possible confounders, i.e. age (mean = 12.56; SD = 0.49), sex (50.4% female), socioeconomic background (parental education and income), marital status, and ethnicity, were assessed at baseline. The level of parental education was measured in years of education, ranging from 6 to 19 years (mean = 13.69; SD = 3.54). Parental income was measured in 2003 (1999/2000 data not available) with the equivalised household income of the child’s mother (expressed as percentile score: mean = 55.67; SD = 26.09). The income of the mother included fewer missing values compared to fathers’ income; it was assumed that children of divorced parents are more likely to live with their mother. Using information on the country of birth of parents and participants, ethnicity was categorised into native Dutch (82.4%), non-Western (10.7%: Turkish, African, Asian and Latin-American) or Western (6.6%: European (excluding The Netherlands and Turkey), North American, Oceanic, Japanese and Indonesian) (Alders 2001). Marital status was categorised into married (86%) or non-married.

### Statistical analysis

Pearson correlations of parental income and parental education with the five personality traits were computed. First, we examined the cross-sectional association of the participant’s income and education with the four health care use outcomes in 2011/2012 (when they were young adults). This was done with cross-tabulations and the corresponding *χ* ^2^-tests. Second, logistic regression was used to examine whether and how personality in 2000 was related to subsequent socioeconomic and health care-related outcomes in 2011/2012. Third, logistic regression analyses estimated the cross-sectional odds ratios (OR) [and 95% confidence intervals (CI)] of participants’ socioeconomic differences in health care use in 2011/2012 (by relating final education and own income to all four health outcomes). In subsequent models, we examined whether the odds ratios decreased, when controlled for personality. All logistic regression analyses were adjusted for all covariates. Sensitivity analyses (including linear regressions) were done using the continuous variants of personality traits, socioeconomic status, and health (costs only). Interactions of final education, own income, parental education and parental income with personality were also studied. Finally, it was also checked whether not controlling for marital status changed our findings.

## Results

Pearson correlations of parental education and income with participants’ personality traits (as continuous variables) were statistically significant, but below |0.10| (not tabulated). Table 1 shows the distribution of participants with high health care use by levels of high, medium and low final education and income in 2011/2012. Hospital admission had the fewest participants (on average 4.9% in each group) compared with medication use (16.5%), high GP costs (18.9%) and HA costs (19.3%).

**Table 1:** Percentages of high health care use by participants’ final educational and income level (The Netherlands, 1999–2012)

*N*		Hospital admission (no, yes)	High GP costs (no, yes)	High hospital costs (no, yes)	Medication use (no, yes)
Final education					
High	5744	192 (3.3%)	891 (15.5%)	911 (15.9%)	852 (14.8%)
Medium	3130	170 (5.4%)	616 (19.7%)	635 (20.3%)	544 (17.4%)
Low	4058	277 (6.8%)	938 (23.1%)	954 (23.5%)	736 (18.1%)
Income					
High	4542	158 (3.5%)	737 (16.2%)	752 (16.6%)	683 (15.0%)
Medium	4416	265 (6.0%)	887 (20.1%)	913 (20.7%)	742 (16.8%)
Low	3974	216 (5.4%)	821 (20.7%)	835 (21.0%)	707 (17.8%)

Table 2 shows that low emotional stability was consistently related to high use of health care and poor socioeconomic outcomes. Low emotional stability was not only associated with lower final education (OR = 1.45; 95% CI 1.27, 1.64), but also with higher GP costs (OR = 1.21; 95% CI 1.08, 1.35), higher hospital costs (OR = 1.23; 95% CI 1.10, 1.37) and higher medication use (OR = 1.39; 95% CI 1.23, 1.56). Participants who were characterised by low openness to experience had a lower odds of both a lower income (OR = 0.78; 95% CI 0.69, 0.87) and a higher use of medication (OR = 0.86; 95% CI 0.47, 0.74). The other personality traits were not related to any of the outcomes (extraversion) or were only related to the socioeconomic outcomes (conscientiousness, agreeableness).

**Table 2:** Odds ratios (95% confident interval) of a low socioeconomic position and high health care use by personality traits, adjusted for age, sex, ethnicity, parents’ marital status, and parental education and income (The Netherlands, 1999–2012)

	Low final education	Low income	High hospital admission	High GP costs	High hospital costs	Medication use
	*N* = 12,274	*N* = 12,538	*N* = 12,932	*N* = 12,932	*N* = 12,932	*N* = 12,932
Conscientiousness						
Medium^a^	**1.27 (1.12, 1.44)**	0.99 (0.88, 1.11)	0.92 (0.76, 1.13)	0.96 (0.86, 1.08)	0.97 (0.87, 1.08)	0.96 (0.86, 1.08)
Low^a^	**1.56 (1.37, 1.77)**	**1.13 (1.02, 1.27)**	1.09 (0.90, 1.33)	1.03 (0.92, 1.15)	1.03 (0.92, 1.15)	0.96 (0.86, 1.08)
Extraversion						
Medium^a^	0.91 (0.79, 1.03)	1.07 (0.96, 1.19)	**0.79 (0.65, 0.97)**	1.05 (0.94, 1.17)	1.04 (0.93, 1.16)	0.95 (0.84, 1.07)
Low^a^	1.02 (0.89, 1.16)	1.10 (0.99, 1.24)	0.94 (0.76, 1.15)	1.07 (0.96, 1.20)	1.05 (0.94, 1.17)	1.06 (0.94, 1.19)
Agreeableness						
Medium^a^	1.06 (0.93, 1.22)	0.98 (0.88, 1.09)	0.86 (0.71, 1.04)	0.95 (0.86, 1.06)	0.95 (0.85, 1.05)	0.92 (0.82, 1.03)
Low^a^	**1.55 (1.36, 1.77)**	**0.84 (0.74, 0.94)**	1.04 (0.85, 1.27)	0.97 (0.87, 1.09)	0.96 (0.86, 1.08)	0.96 (0.84, 1.08)
Emotional stability						
Medium^a^	**1.15 (1.01, 1.31)**	0.98 (0.88, 1.09)	0.99 (0.81, 1.22)	0.96 (0.85, 1.07)	0.97 (0.87, 1.09)	1.08 (0.96, 1.23)
Low^a^	**1.45 (1.27, 1.64)**	1.05 (0.93, 1.17)	**1.24 (1.02, 1.52)**	**1.21 (1.08, 1.35)**	**1.23 (1.10, 1.37)**	**1.39 (1.23, 1.56)**
Openness to experience						
Medium^a^	1.02 (0.90, 1.16)	**0.82 (0.74, 0.92)**	1.01 (0.83, 1.23)	1.07 (0.96, 1.19)	1.04 (0.94, 1.16)	0.96 (0.85, 1.08)
Low^a^	1.05 (0.93, 1.19)	**0.78 (0.69, 0.87)**	1.00 (0.82, 1.22)	0.93 (0.83, 1.04)	0.92 (0.82, 1.03)	**0.86 (0.77, 0.97)**

Bold values indicate significant odd ratios with *p* ≤ 0.05

^a^The reference category “high” personality scores equals the OR of 1.00 and has been left out of the table to present a clearer overview

In 2011/2012, the participant’s low socioeconomic position was associated with all adverse health care outcomes in the same year (Table 3, model 0). The highest ORs were found for hospital admissions. Low final education and low income increased the odds of hospital admission by 2.21 (95% CI 1.81, 2.69) and 1.54 (95% CI 1.25, 1.91), respectively. ORs for low education and low income, associated with medication use, were the smallest and mostly nonsignificant values (OR: 1.39, CI 1.24, 1.57 and OR: 1.13, CI 0.99, 1.27, respectively). The associations with income were generally somewhat smaller and less dose response-like. Comparing model 0 (unadjusted for personality) with models 1–6 (adjusted for the respective personality traits and all traits simultaneously) indicates that ORs relating to the young adults’ socioeconomic differences in health care use in 2011/2012 hardly changed when controlled for personality (as measured in 2000).

**Table 3:** Odds ratios (95% confident interval) of socioeconomic differences in health care use, unadjusted (model 0) and adjusted (model 1–6) for personality traits (all models were adjusted for age, sex, ethnicity, parents’ marital status, and parental education and income) (*n* = 12,932) (The Netherlands, 1999–2012)

	Hospital admissions (no, yes)		High GP costs (no, yes)		High hospital costs (no, yes)		Medication use (no, yes)	
	Medium^a^	Low	Medium^a^	Low	Medium^a^	Low	Medium^a^	Low
Final education								
Model 0 unadjusted for personality	**1.68 (1.36, 2.08)**	**2.21 (1.81, 2.69)**	**1.32 (1.18, 1.49)**	**1.63 (1.46, 1.82)**	**1.34 (1.19, 1.50)**	**1.63 (1.46, 1.81)**	**1.25 (1.10, 1.41)**	**1.39 (1.24, 1.57)**
Model 1 adjusted for conscientiousness	**1.68 (1.35, 2.08)**	**2.20 (1.81, 2.69)**	**1.32 (1.18, 1.49)**	**1.63 (1.47, 1.82)**	**1.34 (1.19, 1.50)**	**1.63 (1.46, 1.82)**	**1.25 (1.11, 1.41)**	**1.39 (1.24, 1.57)**
Model 2 adjusted for extraversion	**1.68 (1.36, 2.07)**	**2.21 (1.81, 2.69)**	**1.32 (1.18, 1.48)**	**1.63 (1.46, 1.82)**	**1.34 (1.19, 1.50)**	**1.63 (1.46, 1.81)**	**1.25 (1.10, 1.41)**	**1.39 (1.24, 1.57)**
Model 3 adjusted for agreeableness	**1.67 (1.35, 2.07)**	**2.20 (1.80, 2.69)**	**1.33 (1.18, 1.49)**	**1.64 (1.47, 1.83)**	**1.35 (1.19, 1.51)**	**1.64 (1.47, 1.82)**	**1.25 (1.11, 1.41)**	**1.39 (1.24, 1.57)**
Model 4 adjusted for emotional stability	**1.67 (1.35, 2.07)**	**2.19 (1.79, 2.67)**	**1.32 (1.17, 1.48)**	**1.62 (1.45, 1.80)**	**1.33 (1.19, 1.49)**	**1.61 (1.45, 1.79)**	**1.24 (1.09, 1.39)**	**1.37 (1.22, 1.55)**
Model 5 adjusted for openness to experience	**1.68 (1.36, 2.09)**	**2.21 (1.81, 2.70)**	**1.33 (1.19, 1.49)**	**1.64 (1.47, 1.83)**	**1.35 (1.20, 1.52)**	**1.64 (1.47, 1.83)**	**1.26 (1.12, 1.43)**	**1.40 (1.25, 1.58)**
Model 6 adjusted for all personality traits	**1.68 (1.36, 2.08)**	**2.20 (1.80, 2.69)**	**1.33 (1.18, 1.49)**	**1.64 (1.47, 1.83)**	**1.35 (1.20, 1.51)**	**1.64 (1.47, 1.83)**	**1.26 (1.11, 1.42)**	**1.40 (1.25, 1.58)**
Own income								
Model 0 unadjusted for personality	**1.68 (1.37, 2.06)**	**1.54 (1.25, 1.91)**	**1.19 (1.07, 1.34)**	**1.25 (1.12, 1.40)**	**1.21 (1.09, 1.35)**	**1.25 (1.11, 1.39)**	1.04 (0.93, 1.17)	1.13 (0.99, 1.27)
Model 1 adjusted for conscientiousness	**1.67 (1.37, 2.05)**	**1.53 (1.24, 1.89)**	**1.19 (1.07, 1.34)**	**1.25 (1.12, 1.40)**	**1.21 (1.09, 1.35)**	**1,25 (1.11, 1.39)**	1.04 (0.93, 1.17)	1.13 (1.00, 1.27)
Model 2 adjusted for extraversion	**1.68 (1.37, 2.06)**	**1.54 (1.25, 1.91)**	**1.19 (1.07, 1.34)**	**1.25 (1.12, 1.40)**	**1.21 (1.08, 1.35)**	**1.25 (1.11, 1.39)**	1.04 (0.93, 1.17)	1.12 (0.99, 1.27)
Model 3 adjusted for agreeableness	**1.68 (1.37, 2.06)**	**1.55 (1.25, 1.91)**	**1.20 (1.07, 1.34)**	**1.25 (1.12, 1.40)**	**1.21 (1.09, 1.35)**	**1.25 (1.12, 1.39)**	1.04 (0.93, 1.17)	1.13 (1.00, 1.27)
Model 4 adjusted for emotional stability	**1.67 (1.36, 2.04)**	**1.53 (1.23, 1.89)**	**1.19 (1.07, 1.33)**	**1.24 (1.11, 1.39)**	**1.21 (1.08, 1.35)**	**1.24 (1.11, 1.39)**	1.03 (0.92, 1.16)	1.12 (0,99, 1.26)
Model 5 adjusted for openness to experience	**1.68 (1.37, 2.06)**	**1.54 (1.25, 1.91)**	**1.20 (1.08, 1.34)**	**1.25 (1.12, 1.40)**	**1.21 (1.09, 1.35)**	**1.25 (1.11, 1.39)**	1.04 (0.86, 1.08)	1.12 (0.99, 1.27)
Model 6 adjusted for all personality traits	**1.67 (1.36, 2.05)**	**1.54 (1.24, 1.90)**	**1.19 (1.07, 1.33)**	**1.24 (1.11, 1.39)**	**1.21 (1.08, 1.35)**	**1.24 (1.11, 1.39)**	1.04 (0.92, 1.17)	1.12 (0.99, 1.26)

Bold values indicate significant odd ratios with *p* ≤ 0.05

^a^Both final education and income were dichotomised for these analyses (0 = 80% highest education or income versus 1 = 20% lowest education or income)

Sensitivity analyses for an extended period of health care use, by adding data from 2009 and 2010 (which were also available), did not result in different findings. Socioeconomic status of participants and parents did not interact with personality. Using the continuous versions of variables (of personality and costs), including linear regression analyses for the continuous GP and hospital costs, did not result in a different pattern of findings. Finally, not controlling for marital status did not change the findings.

## Discussion

Using a Dutch, large-scale prospective study, personality at the age of 12 was hardly related to the socioeconomic background of the parents. High openness to experience and low emotional stability were related to both later high health care use and later low socioeconomic attainment in young adults. However, most likely due to these underlying associations being too small, we could not find proof for personality as a driving force for socioeconomic differences in young adults’ health care use. Unexpectedly, this study therefore does not support the findings of Nabi et al. (2008) and Chapman et al. (2009); they reported an attenuated effect of socioeconomic position on mortality after controlling for personality.

The strength of our study is the use of the 100-item FFPI and the specifics of the design enabling us to have personality measured prior to participants’ later socioeconomic achievements and later health care use. Compared to previous studies (Chapman et al. 2009; Nabi et al. 2008; Roberts et al. 2007; Singh-Manoux 2005; Van Bon-Martens et al. 2012), this may have allowed a more valid examination of the causal role that personality might play in generating socioeconomic differences in health care use, particularly in a life phase where important processes of social mobility take place. Our study certainly also has its limitations. First, use of health services was measured rather than health per se. It is not unlikely that certain personality characteristics, even with the same type and severity of disease, might increase the probability of looking for medical help and actually getting the health care services (Maier 2006; Olsson and Dahl 2009; Ten Have et al. 2005). Hence, it is important to frame our findings in terms of health care use and medical consumption rather than health per se. Second, all of our health care outcomes have their own advantages and disadvantages. Lack of complete coverage is a concern, particularly for the hospital admission data where the coverage was 84%. Furthermore, regarding the linking procedures, it had to be assumed that those without a registered health care use had “good” health (no health care use) outcomes, while some of the initial cohort might have had incident health problems and related medical consumption that was not registered, e.g. because they had moved abroad. Despite differences in what they measure (sometimes subtle, as with hospital admission and costs) and despite their specific advantages and disadvantages, findings across the four health care use outcomes were very similar. Third, experts are not unanimous about at what age personality is fully developed and about how trait-like personality actually is (Edmonds et al. 2008; Roberts et al. 2006). It is unclear how this might have affected our findings. Hence, a repeated later personality assessment would have been useful to check the consistency of the personality traits. Similarly, also a longer follow-up would have been useful, looking beyond young adulthood when more (severe) health problems would have occurred (Edmonds et al. 2008). Finally, personality had many missing scores (23%). However, comparing relevant variables of participants with and without missing personality scores showed slight differences (not tabulated). More missing values on personality traits occurred in lower educated participants (about 8% difference), participants with lower socioeconomic backgrounds of their parents (about 3.8%), and participants with lower incomes (about 2.5%). This caused the remaining sample to be slightly higher in socioeconomic characteristics. It is unclear how that exactly may have affected our findings.

In conclusion, we may say that our results in Dutch young adults, when it regards personality, do not support the so called “indirect selection” theory on socioeconomic health differences. From that theory, it could have been expected that “adverse” personality characteristics are causally fundamental in the development of socioeconomic health differences. Our findings thus do not help in better explaining socioeconomic differences in health care use, nor do they help in envisaging different types of interventions aimed at tackling these differences. Future studies should try to avoid our study’s drawbacks and evaluate how also other third factors, such as control beliefs (Bosma et al. 2014) and intellectual abilities (Batty et al. 2006; Mackenbach 2005), might be driving forces in the aetiology of socioeconomic health differences.
